# Unilateral Vocal Cord Paralysis Caused by Cervical Esophageal Duplication Cyst Containing a Foreign Body: A Case Report

**DOI:** 10.70352/scrj.cr.25-0362

**Published:** 2025-09-30

**Authors:** Yoshiko Usui, Katsuhisa Baba, Yuki Tsuji, Kyosuke Kuramochi, Shuto Fujihara, Tatsuki Kosaka, Waka Nakata, Mari Shimada, Makoto Ito, Keita Terui

**Affiliations:** 1 Department of Pediatric Surgery, Jichi Children’s Medical Center Tochigi, Jichi Medical University School of Medicine, Shimotsuke, Tochigi, Japan; 2 Department of Diagnostic Pathology, Jichi Medical University Hospital, Shimotsuke, Tochigi, Japan; 3 Department of Pediatric Imaging, Jichi Children’s Medical Center Tochigi, Jichi Medical University School of Medicine, Shimotsuke, Tochigi, Japan; 4 Department of Pediatric Otolaryngology, Jichi Children’s Medical Center Tochigi, Jichi Medical University School of Medicine, Shimotsuke, Tochigi, Japan

**Keywords:** cervical esophageal duplication, foreign body, granuloma, cervical abscess, unilateral vocal cord paralysis, recurrent laryngeal nerve paralysis

## Abstract

**INTRODUCTION:**

Cervical esophageal duplication is a rare congenital anomaly that occasionally causes compressive symptoms. Herein, we present a unique case of unilateral vocal cord paralysis caused by a cervical esophageal duplication cyst with a granuloma containing a foreign body.

**CASE PRESENTATION:**

A 10-year-old boy presented with a 1.5-month history of hoarseness and choking. Laryngoscopy revealed left vocal cord paralysis, and CT revealed a mass near the inferior pole of the left thyroid lobe. Fine-needle aspiration cytology revealed no evidence of malignancy. Ultrasonography and MRI findings suggested an esophageal duplication cyst with an inflammatory granuloma. Antibiotic therapy was ineffective, and a surgical approach was considered. The esophageal duplication cyst was resected through a collar incision, and a film-like foreign body with granulation tissue was extracted from the abscess. The recurrent laryngeal nerve was preserved, and the vocal cord function improved within 2 months.

**CONCLUSIONS:**

This case highlights the unusual presentation of an inflamed cervical esophageal duplication cyst with an embedded foreign body, which resulted in recurrent laryngeal nerve impairment and subsequent unilateral vocal cord paralysis. Early surgical intervention with careful nerve preservation can result in a functional recovery.

## INTRODUCTION

Esophageal duplications are rare congenital anomalies that most commonly occur in the thoracic esophagus. Isolated cervical esophageal duplications are rare. Alimentary tract duplications have an estimated incidence of 1 in 4500 in autopsy series, with approximately 1% occurring in the cervical region, as reported in a meta-analysis.^1)^ The location and size of the duplications determine the clinical presentation. Most cervical esophageal duplications present in the first year of life, typically with respiratory problems, feeding difficulties, or a palpable neck mass.^2)^ Herein, we present a unique case of unilateral vocal cord paralysis caused by a cervical esophageal duplication cyst with a granuloma containing a foreign body. To the best of our knowledge, this is the first reported case of an inflamed cervical esophageal duplication resulting in recurrent laryngeal nerve impairment and subsequent unilateral vocal cord paralysis, with postoperative recovery of nerve function.

## CASE PRESENTATION

**Fig. 1:**
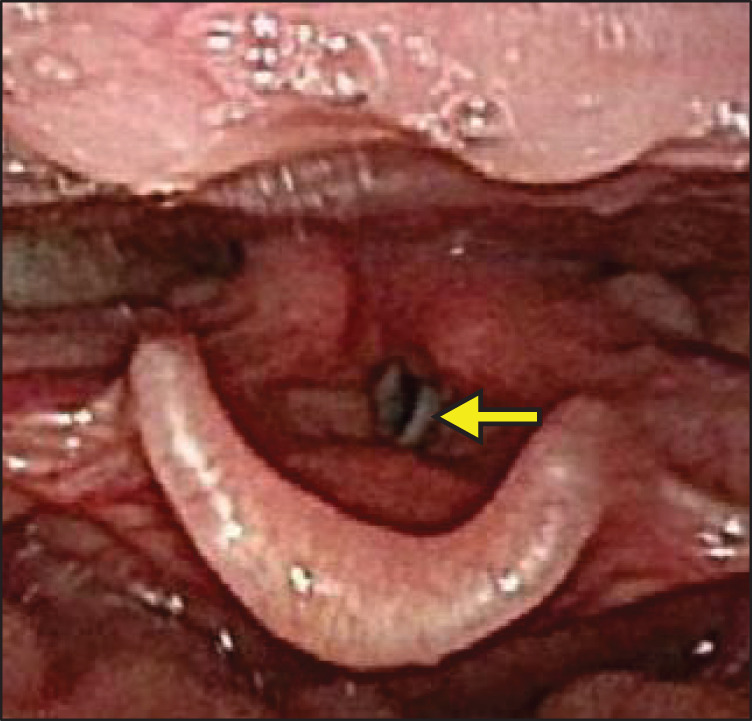
Laryngoscopy showing left vocal cord paralysis with lateral fixation (arrow).

**Fig. 2:**
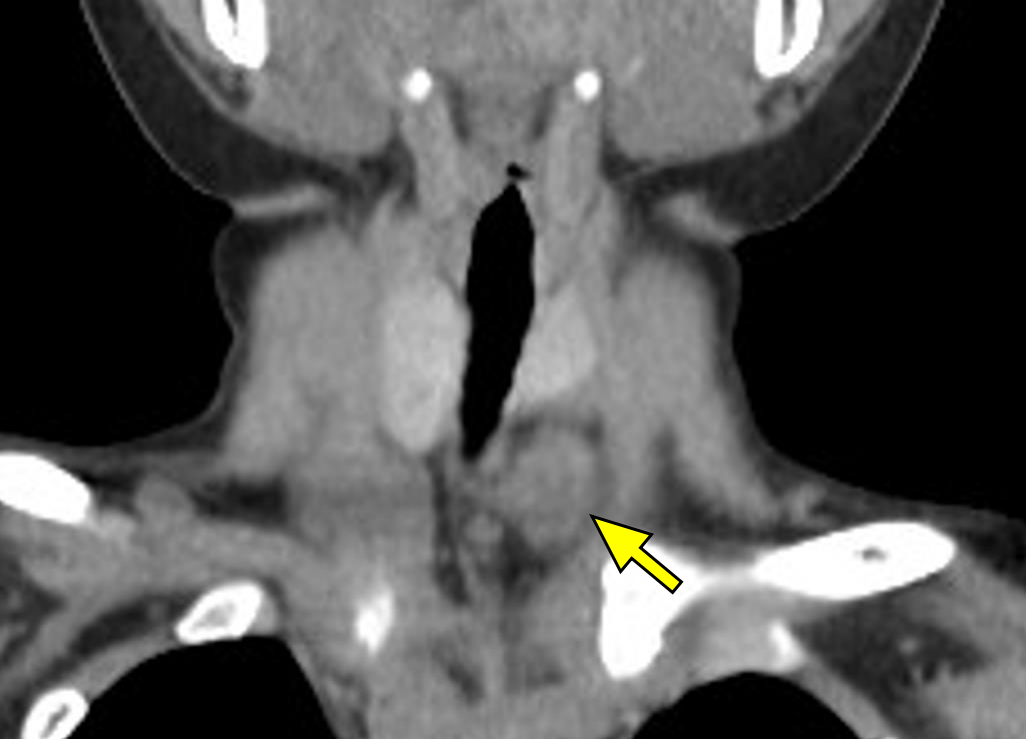
Preoperative contrast-enhanced CT scan showing a 4-cm mass (arrow) near the inferior pole of the left thyroid lobe.

**Fig. 3:**
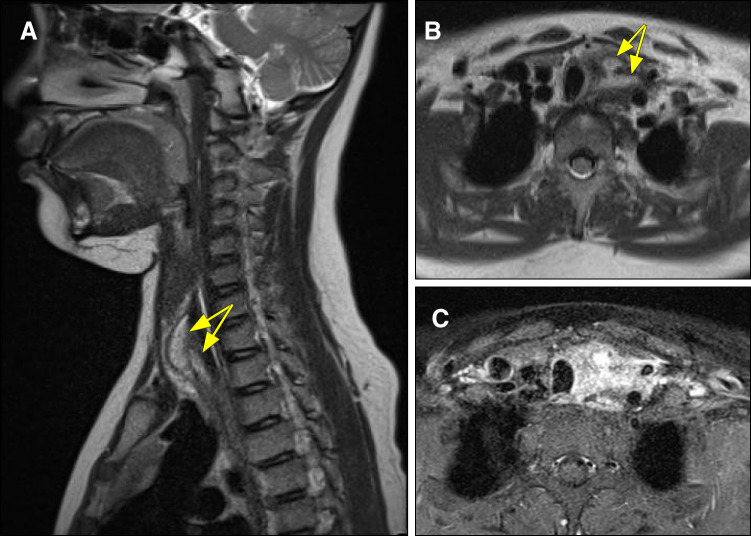
MRI findings. (**A**, **B**) T1-weighted enhanced MRI demonstrating a 22 × 20 × 46-mm cyst with constriction (arrows). (**C**) Contrast-enhanced MRI showing extensive peripheral enhancement around the cyst.

**Fig. 4:**
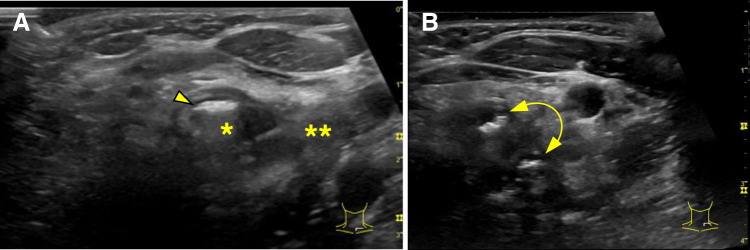
US findings. (**A**) US demonstrating a cystic mass consisting of 2 cavities. The cephalad cyst (asterisk) contains internal air (arrowhead) and has a wall structure resembling those of the gastrointestinal tract. The caudal mass (double asterisk) is filled with highly viscous fluid. (**B**) Air passage between the cyst and the normal esophagus (bidirectional arrow). US, ultrasonography

A previously healthy 10-year-old boy with a 1.5-month history of hoarseness and choking was referred to our hospital. At another hospital, left vocal cord paralysis with lateral fixation was noted; however, no improvement was observed after 1 month (**Fig. 1**). Contrast-enhanced CT revealed a 4-cm mass near the inferior pole of the left thyroid lobe (**Fig. 2**). The mass was not discernible upon visual inspection or palpation, and no compression of the airway or esophagus was observed upon imaging. Fine-needle aspiration cytology yielded negative results. MRI demonstrated a 22 × 20 × 46-mm cyst with a constricted portion (**Fig. 3A** and **3B**) and peripheral enhancement (**Fig. 3C**), but no significant diffusion restriction. An inflammatory granuloma, potentially associated with either an esophageal duplication cyst or esophageal diverticulum, was suspected. No malignant findings were observed. A 2-week course of antibiotic therapy was ineffective, and the vocal cord paralysis persisted. However, with no progression of symptoms, daily life was not significantly affected except for the inability to speak loudly. Ultrasonography demonstrated the mass as a lobulated cyst with a wall structure resembling those of the gastrointestinal tract (**Fig. 4A**) and an air passage between the cyst and the normal esophagus (**Fig. 4B**), strongly indicative of an esophageal duplication cyst. However, esophagography did not reveal contrast medium entering the cyst. No findings suggestive of a pyriform sinus fistula were observed. A collaborative surgical procedure involving the pediatric surgery team and the otolaryngology team was performed 1 month after the definitive diagnosis of esophageal duplication with an associated granuloma.

**Fig. 5:**
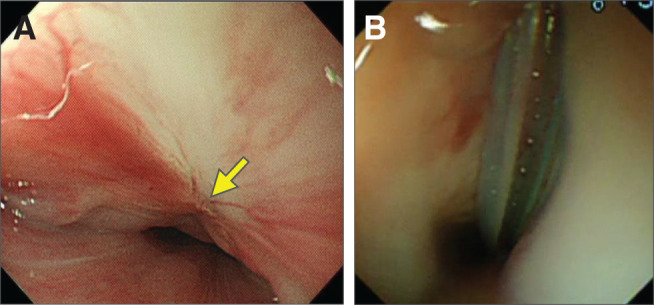
Upper gastrointestinal endoscopy findings. (**A**) Preoperative upper gastrointestinal endoscopy showing a focal mucosal irregularity with a recess, suspected to represent a communication site with the cyst. No visible opening was found, and catheterization of the cyst was unsuccessful. (**B**) Following excision of the esophageal duplication cyst and removal of the foreign body with associated granulation tissue, an attempt to insert a drain into the abscess cavity resulted in the drain tip entering the normal esophageal lumen through the area indicated in (**A**).

**Fig. 6:**
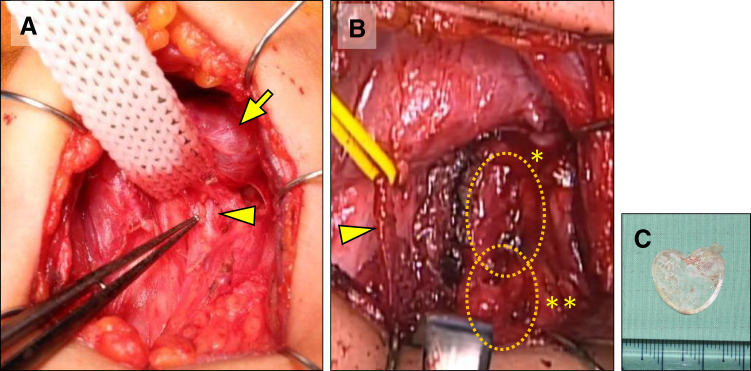
Surgical findings. (**A**) The recurrent laryngeal nerve (arrowhead) is identified dorsal to the inferior pole of the left thyroid lobe (arrow). (**B**) The cystic mass, located posterior to the recurrent laryngeal nerve (arrowhead), comprises the cephalad esophageal duplication cyst (dotted circle with an asterisk) and a caudal abscess cavity filled with granulation tissue (dotted circle with a double asterisk). (**C**) A heart-shaped film-like foreign body was extracted from the abscess.

During surgery, upper gastrointestinal endoscopy revealed a focal mucosal irregularity with a recess, suspected to represent the site of communication with the cyst. However, no overt opening was visualized, and catheterization of the cyst was unsuccessful (**Fig. 5A**). A collar incision was made on the left side of the neck to access the mass. Dense fibrous adhesions were observed adjacent to the thyroid gland. The recurrent laryngeal nerve was identified dorsal to the inferior pole of the left thyroid lobe (**Fig. 6A**) and was carefully dissected free from the mass. The mass consisted of a cephalad esophageal duplication cyst and a caudal abscess cavity filled with granulation tissue (**Fig. 6B**). A film-like foreign body with granulation tissue was extracted from the abscess (**Fig. 6C**). After removing most of the duplication cyst, the mucosa at the common wall—sharing the muscular layer with the normal esophagus—was resected. Communication between the cystic mass and the normal esophagus became evident when an attempt to insert a drain into the abscess cavity resulted in the drain tip entering the normal esophageal lumen (**Fig. 5B**). The esophageal wall was subsequently sutured under upper gastrointestinal endoscopic guidance, and the drain was placed into the abscess cavity.

**Fig. 7:**
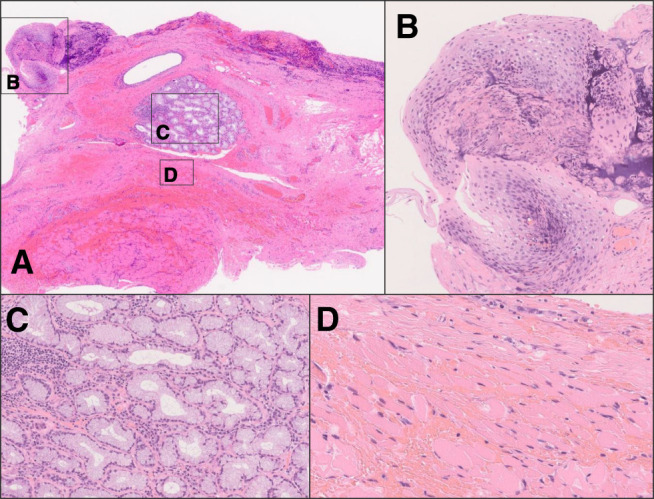
Histopathological findings. (**A**) The duplicated esophageal wall contains the stratified squamous epithelium (**B**), esophageal glands (**C**), striated muscle fibers (**D**), and smooth muscle fibers. Proliferative granulation tissue with neovascularization was also observed. No malignancies were detected.

The patient was extubated on POD 2, and oral intake was resumed on day 10 without signs of aspiration. He was able to speak in a loud voice early in the postoperative course. The abscess resolved following drainage. On POD 5, the left vocal cord was fixed at the midline, but glottic closure was satisfactory. By POD 54, vocal cord mobility had nearly returned to normal. Neither the patient nor their family had any recollections of heart-shaped film-like foreign objects. Histopathological examination (**Fig. 7**) revealed that the duplicated esophageal wall contained striated muscle fibers, smooth muscle fibers, and esophageal glands, and the epithelium consisted of stratified squamous cells. Proliferative granulation tissue with neovascularization was also observed. No malignancies were detected.

## DISCUSSION

In this case, surgical intervention—including excision of the esophageal duplication cyst, removal of the foreign body with granulation tissue, and drainage of the abscess—resulted in a rapid improvement of vocal cord mobility. Although the cervical duplication cyst and associated granuloma were diagnosed preoperatively, the foreign body was not visualized on imaging due to its thin, film-like appearance. In general, diagnosing esophageal foreign bodies that have migrated outside the esophagus is challenging. In a case similar to ours, a foreign body was reported within an esophageal duplication cyst, where communication between the cyst and the normal esophagus was not obvious during surgery. The foreign body was a bingo chip, likely ingested approximately 18 months before surgery.^3)^ Esophageal foreign bodies can occasionally migrate through the esophageal wall into the adjacent tissues, resulting in delayed symptoms.^4,5)^ Additionally, a foreign body granuloma mimicking a subepithelial neoplasm has also been reported in the literature.^6)^

It was difficult to explain the association between the esophageal duplication cyst and unilateral laryngeal nerve paralysis before the surgery. Although several reports have described large esophageal duplications causing respiratory distress due to direct compression of the trachea^2,7)^ or recurrent laryngeal nerve,^8)^ the duplication cyst in the present case was relatively small and did not cause airway stenosis or esophageal obstruction. Recurrent laryngeal nerve paralysis was likely caused not only by direct mechanical compression but also by chronic inflammatory changes induced by the foreign body. Because recurrent laryngeal nerve paralysis secondary to an inflamed cervical esophageal duplication has not been previously reported, chronic inflammation due to a foreign body was not initially suspected. While a previous report on carcinoma arising from an esophageal duplication cyst in a young adult exists,^9)^ the possibility of malignancy could not be ruled out.

Unilateral vocal cord paralysis without laryngeal lesions is a relatively common clinical presentation. It can be a manifestation of numerous diseases originating in the thorax, head, neck, or systemic diseases. The most common extralaryngeal cause of vocal cord paralysis is cervical surgery, followed by tumors and idiopathic paralysis.^10)^ In the pediatric population, the leading cause of unilateral vocal cord paralysis is trauma resulting from cardiac surgery, followed by idiopathic paralysis.^11)^ Dysphonia is often the 1st symptom of these diseases, and it is a warning sign that leads to a diagnosis of the underlying pathology.^10)^

Preoperative vocal cord paralysis in patients with thyroid disease is believed to be associated with malignancy. In malignant diseases, recurrent laryngeal nerve paralysis can be caused by the direct invasion of malignant cells or nodular compression.^12)^ However, benign thyroid lesions can also cause recurrent laryngeal nerve paralysis through mechanisms such as compression, stretching, inflammation, or edema^13)^; the recurrent laryngeal nerve has a good chance of functional recovery after its identification and preservation during operation.^14,15)^ Furthermore, postoperative recovery of vocal cord function is thought to be largely dependent on the duration of vocal cord paralysis.^13)^

When symptoms of vocal cord paralysis in children with a cervical mass persist or fail to improve with medical therapy, rare malignancies and granulomas with foreign bodies should be included in the differential diagnosis. Prolonged deep cervical inflammation can lead to chronic perineural fibrosis,^13)^ making timely surgical intervention even more critical in preventing prolonged recurrent laryngeal nerve damage.

## CONCLUSIONS

We report a rare pediatric case of unilateral vocal cord paralysis resulting from recurrent laryngeal nerve impairment caused by a cervical esophageal duplication cyst with a granuloma containing a foreign body. Although medical therapy was insufficient to resolve the associated cervical granuloma, surgical excision of the lesions led to a rapid improvement in vocal cord mobility. Timely examination and surgical intervention are crucial for the recovery from vocal cord paralysis.

## DECLARATIONS

### Availability of data and materials

Data sharing is not applicable to this article as no datasets were generated or analyzed during the study.
